# Synthetic Fluororutaecarpine Inhibits Inflammatory Stimuli and Activates Endothelial Transient Receptor Potential Vanilloid-Type 1

**DOI:** 10.3390/molecules22040656

**Published:** 2017-04-19

**Authors:** Chi-Ming Lee, Jiun-An Gu, Tin-Gan Rau, Chi Wang, Chiao-Han Yen, Shih-Hao Huang, Feng-Yen Lin, Chun-Mao Lin, Sheng-Tung Huang

**Affiliations:** 1 Graduate Institute of Medical Sciences, College of Medicine, Taipei Medical University, Taipei 11031, Taiwan; januslee@tmu.edu.tw (C.-M.L.); chiw@tmu.edu.tw (C.W.); sausage0419@gmail.com (C.-H.Y.); 2 Institute of Chemical Engineering, College of Engineering, National Taipei University of Technology, Taipei 10608, Taiwan; b883038@hotmail.com (J.-A.G.); rautnn@gmail.com (T.-G.R.); 3 Department of Food and Beverage Management, Taipei College of Maritime Technology, Taipei 11174, Taiwan; f0711@mail.tcmt.edu.tw; 4 Department of Internal Medicine, School of Medicine, College of Medicine, Taipei Medical University, Taipei 11031, Taiwan; g870905@tmu.edu.tw; 5 Department of Biochemistry, School of Medicine, College of Medicine, Taipei Medical University, Taipei 11031, Taiwan; 6 Institute of Biochemical and Biomedical Engineering, College of Engineering, National Taipei University of Technology, Taipei 10608, Taiwan

**Keywords:** inflammation, fluoro-rutaecarpine, iNOS, TRPV-1, eNOS

## Abstract

The natural product, rutaecarpine (RUT), is the main effective component of *Evodia rutaecarpa* which is a widely used traditional Chinese medicine. It has vasodilation, anticoagulation, and anti-inflammatory activities. However, further therapeutic applications are limited by its cytotoxicity. Thus, a derivative of RUT, 10-fluoro-2-methoxyrutaecarpine (F-RUT), was designed and synthesized that showed no cytotoxicity toward RAW264.7 macrophages at 20 μM. In an anti-inflammation experiment, it inhibited the production of nitric oxide (NO) and tumor necrosis factor (TNF)-α in lipopolysaccharide (LPS)-stimulated RAW264.7 macrophages; cyclooxygenase (COX)-2 and inducible NO synthase (iNOS) induced by LPS were also downregulated. After 24 h of treatment, F-RUT significantly inhibited cell migration and invasion of ovarian A2780 cells. Furthermore, F-RUT promoted expressions of transient receptor potential vanilloid type 1 (TRPV1) and endothelial (e)NOS in human aortic endothelial cells, and predominantly reduced the inflammation in ovalbumin/alum-challenged mice. These results suggest that the novel synthetic F-RUT exerts activities against inflammation and vasodilation, while displaying less toxicity than its lead compound.

## 1. Introduction

Endothelial cells (ECs) of arteries are important for the trafficking of nutrients and participate in many physiologic events, such as inflammation and angiogenesis. Atherosclerosis is primarily associated with a series of reactions within the tunica intima and involves monocyte recruitment, macrophage formation, lipid accumulation, extracellular matrix (ECM) production, and smooth muscle cell migration [1]. Compounds with inhibitory effects on vascular inflammation and cell migration would be beneficial for antiatherogenic progression [2].

Rutaecarpine (RUT) is one of the main bioactive ingredients extracted from the traditional medicine *Evodia rutaecarpa* [3] and exhibits a wide spectrum of biological activities [4]. RUT can improve atherosclerosis by preventing monocyte adhesion to the vascular endothelium [5]. RUT reduced the prostaglandin production of lipopolysaccharide (LPS)-activated RAW264.7 macrophages, but did not affect levels of cyclooxygenase (COX)-2 messenger (m)RNA or protein [6]. The vasorelaxant effect of RUT in isolated mesenteric arteries of rats was reported to be associated with Ca^2+^ flux activity [7,8]. RUT lowered blood pressure through the endothelial Ca^2+^-nitric oxide (NO)-cGMP pathway to reduce residual muscle tension [9]. The calcitonin gene-related peptide (CGRP), a major neurotransmitter produced in peripheral and central neurons, plays a key role in maintaining endothelial homoeostasis. Decreased plasma CGRP levels cause cardiac susceptibility to ischemia-reperfusion injury, and RUT reverses that decrease by stimulating CGRP production [10,11]. CGRP counteracts angiotensin (Ang) II-induced endothelial progenitor cell senescence by suppressing reactive oxygen species (ROS) and NADPH oxidase [12].

Activation of transient receptor potential vanilloid type 1 (TRPV1) in ECs may protect against cardiovascular diseases such as hypertension and stroke [13,14]. Release of CGRP by activation of vanilloid receptors plays an important role in the vasodilation effects of RUT [15,16]. NO released by activation of endothelial NO synthase (eNOS) leads to vascular relaxation mediated by CGRP and TRPV1 stimulation [17]. TRPV1-dependent atheroprotection was demonstrated in mice [18]. RUT was reported to be a potential therapeutic agent for arterial thrombosis because of its antiplatelet effect in vivo [19,20]. Alkaloid compounds also showed anticancer activities by inducing cell cycle arrest or apoptosis in vitro and in vivo [21,22]. RUT showed high toxicity to lymphoblasts and inhibited ATP-dependent efflux pumps in a blood-brain barrier model with porcine brain capillary ECs [23], that thus restricts its application in vascular diseases. A variety of structural modifications of natural products were designed and synthesized for better biological applications [24]. RUT derivatives were designed and synthesized to activate TRPV1 for enhanced vasodilator and hypotensive effects. The 14-N atom of RUT is critical for its activity [25]. Bromo-rutaecarpine was designed to broaden the potential for application. However, the bromo-derivative is less stable than fluoro-derivatives due to it being more bulky in substitution [26]. Analogs of RUT synthesized for this study exhibited very low cytotoxicity, but had anti-inflammatory activity and TRPV1-upregulating effects. The results provide insights into the use of the TRPV1 agonist, F-RUT, in vascular diseases.

## 2. Results

### 2.1. Design and Synthesis of F-RUT

**Scheme 1:**
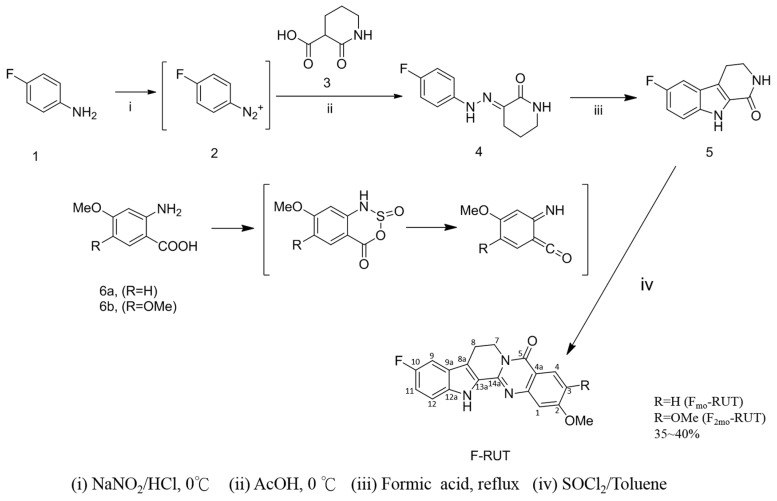
Synthesis of 10-fluoro-2-methoxyrutaecarpine (F-RUT) and 10-fluoro-2,3-dimethoxyrutaecarpine.

The synthetic schemes for preparing F-RUT derivatives are outlined in Scheme 1. The main intermediate, substituted carboline (**5**) was prepared from substituted aniline following the procedure described by Narayanan and Tschesche [27,28]. Aniline (**1**) was subjected to the Sandmeyer reaction to give the diazonium salt, which was then coupled to a carboxylic acid **3** to yield hydrazone (**4**), and treatment of the hydrazone (**4**) in an acidic condition gave the carboline (**5**) (Supplementary Figure S1) in a 58% yield (three steps). The carboline (**5**) was then coupled with in-situ activated substituted *o*-aminobenzoic acid derivatives (**6a**,**b**), which were pretreated with thionyl chloride in the presence of toluene at 70–80 °C to provide 10-fluoro-2-methoxyrutaecarpine (F-RUT) and 10-fluoro-2,3-dimethoxyrutaecarpine in 35% and 40% overall yields (four steps), respectively. The synthetic products were identified by ^1^H and ^13^C nuclear magnetic resonance (NMR), infrared (IR), and mass spectrometry (MS). For F-RUT, FT-IR (KBr, cm^−1^): 3347 (N-H) and 1652 (carbonyl group) (Supplementary Figure S2). ^1^H-NMR (CDCl_3_, ppm): δ 3.18 (t, *J* = 6.9 Hz, 2H, 2H-8), 4.57 (t, *J* = 6.9 Hz, 2H, H-7), 3.93 (s, 3H, 2-OMe), 7.02 (dd, *J* = 8.9, 2.4, 1H, H-3), 7.05 (d, *J* = 2.4, 1H, H-1), 8.21 (d, *J* = 8.9, 1H, H-4), 7.11 (d, *J* = 8.8, 1H, H-12), 7.38 (d, *J* = 8.8, 1H, H-11), 8.99 (s, 1H, H-9), 12.04 (s, 1H, N-H) (Supplementary Figure S3). MS-ESI (*m/z*) ([M − H]^−^): calcd. 335.3; found 334.4 (Supplementary Figure S4). For 10-fluoro-2,3-dimethoxyrutaecarpine (F_2MO_-RUT), FT-IR (KBr, cm^−1^): 3398 (N-H) and 1641 (carbonyl group) (Supplementary Figure S5). ^1^H-NMR (CDCl_3_, ppm): δ 3.18 (t, *J* = 6.8 Hz, 2H, H-8), 4.57 (t, *J* = 6.8 Hz, 2H, H-7), 3.92 (s, 3H, O-Me), 3.88 (s, 3H, O-Me), 7.04 (s, 1H, H-1), 7.11 (d, *J* = 8.9, 1H, H-12), 7.37 (d, *J* = 8.9, 1H, H-11), 7.66 (s, 1H, H-4), 8.94 (s, 1H, H-9), 11.87 (s, 1H, N-H) (Supplementary Figure S6). MS-ESI (*m/z*) ([M − H]^−^): calcd. 365.3; found 364.4 (Supplementary Figure S7).

### 2.2. NO and TNF-Alpha Releases by LPS-Treated Macrophages Were Suppressed by F-RUT

**Figure 1:**
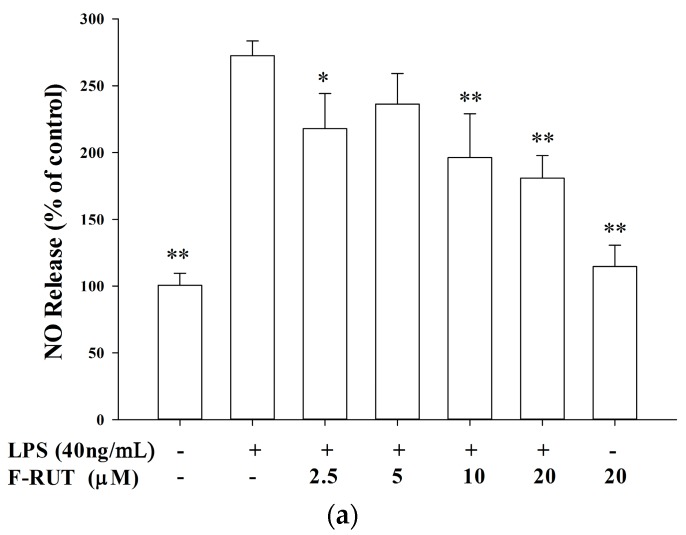

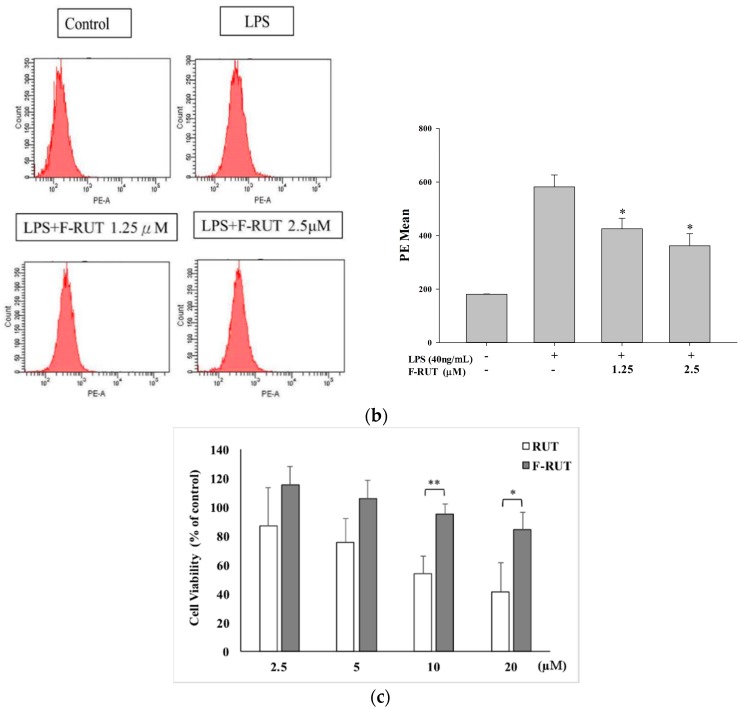
Effects of 10-fluoro-2-methoxyrutaecarpine (F-RUT) on nitric oxide (NO) and tumor necrosis factor (TNF)-α release by lipopolysaccharide (LPS)-treated (40 ng/mL) RAW264.7 macrophages. (**a**) NO levels were detected in culture medium using the Griess reaction; (**b**) TNF-α release in cell supernatants was detected using a mouse TNF-α Quantikine kit; (**c**) Cell viability upon F-RUT and rutaecarpine (RUT) treatment for 24 h in an MTT assay. Values are expressed as the mean ± SE. * *p* < 0.05, ** *p* < 0.01.

NO production of LPS-treated RAW264.7 macrophages increased compared to that of untreated cells. Co-treatment with the synthesized F-RUT suppressed NO production in a concentration-dependent (0–20 μM) manner (* *p* < 0.05, ** *p* < 0.01, compared to the LPS-treated group) (Figure 1a). A consistent concentration-dependently potent (* *p* < 0.05, compared to the LPS-treated group) (Figure 1b) suppressive effect of TNF-α released into the medium was also shown. The suppressive effects were not due to cytotoxic activity because F-RUT showed no cytotoxicity at concentrations of 0–20 μM (* *p* < 0.05, ** *p* < 0.01, compared to the RUT-treated group) (Figure 1c).

### 2.3. F-RUT Suppresses Inducible (i)NOS and COX-2 Expressions which Was Correlated with Inhibition of Nuclear Factor (NF)-κB Activity in LPS-Activated Macrophages

**Figure 2:**
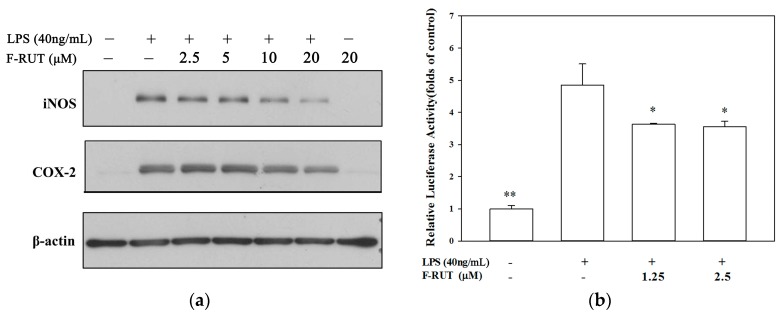
Effect of 10-fluoro-2-methoxyrutaecarpine (F-RUT) on inducible nitric oxide synthase (iNOS) and cyclooxygenase (COX)-2 expressions by lipopolysaccharide (LPS)-treated RAW264.7 macrophages (**a**), and luciferase reporter plasmid-transfected macrophages (**b**). Cells were transfected with 2.5 μg of the pGL4.32 [luc2P/NF-κB-RE/Hygro] reporter plasmid, then treated with different concentrations of F-RUT and LPS (40 ng/mL) for 24 h. Levels of luciferase activity were determined as described in Materials and Methods. Values are expressed as the mean ± SE of triplicate tests. * *p* < 0.05, ** *p* < 0.01 versus LPS treatment.

We further investigated the effect of F-RUT on protein levels of iNOS and COX-2. LPS-treated RAW264.7 macrophages exhibited significantly elevated protein amounts of iNOS and COX-2, while F-RUT suppressed their expressions in a concentration-dependent manner (Figure 2a). β-Actin protein levels of the loading controls remained constant. In the inflammation reaction, NF-κB activation triggers the induction of COX-2 and iNOS. We determined whether F-RUT suppressed NF-κB activation in LPS-activated macrophages. An NF-κB-dependent luciferase reporter plasmid was transiently transfected in LPS-induced macrophages to confirm whether F-RUT inhibited NF-κB-binding activity. F-RUT inhibited LPS-induced NF-κB transcriptional activity at 0–2.5 μM (* *p* < 0.05, ** *p* < 0.01, compared to the LPS-treated group) (Figure 2b). The results suggested that inhibition of iNOS and COX-2 expression by F-RUT was correlated with suppression of NF-κB activation. Compared to RUT, F-RUT showed less cytotoxicity, but retained the anti-inflammatory activity.

### 2.4. F-RUT Inhibited Cell Migration/Invasion

**Figure 3:**
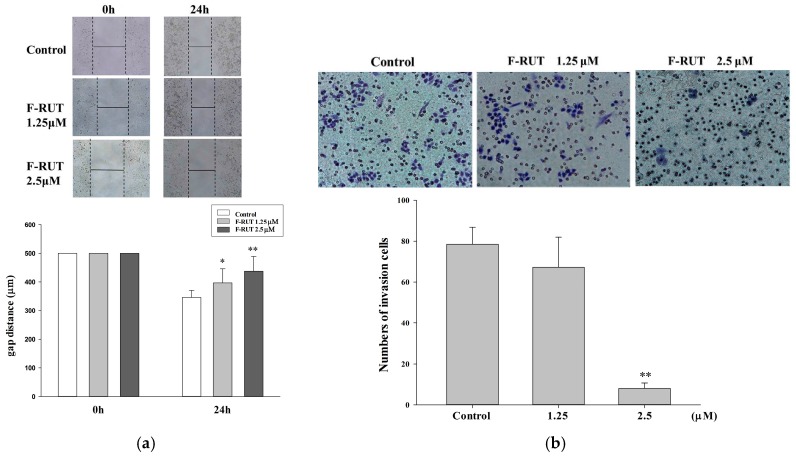
Effects of 10-fluoro-2-methoxyrutaecarpine (F-RUT) on migration and invasion. Cell migration (**a**) and cell invasion (**b**) were detected following F-RUT treatment for 0–24 h, and photographed with a microscope (upper panel). The statistical analysis is shown in the lower panel. (* *p* < 0.05, ** *p* < 0.01).

The effects of F-RUT on inhibiting cell migration and invasion were investigated. As illustrated in Figure 3a, wound-healing assays used an ovarian carcinoma A2780 cell line in the presence of F-RUT (0–5 μM) for 0–24 h. The migration rate was measured using imaging software, and Student’s *t*-test was used for the statistical analysis. F-RUT showed significant effects against cell migration. F-RUT (0–2.5 μM) treatments for 24 h also exhibited invasion inhibitory activity in a transwell assay (Figure 3b).

### 2.5. F-RUT Activated TRPV1 and eNOS

**Figure 4:**
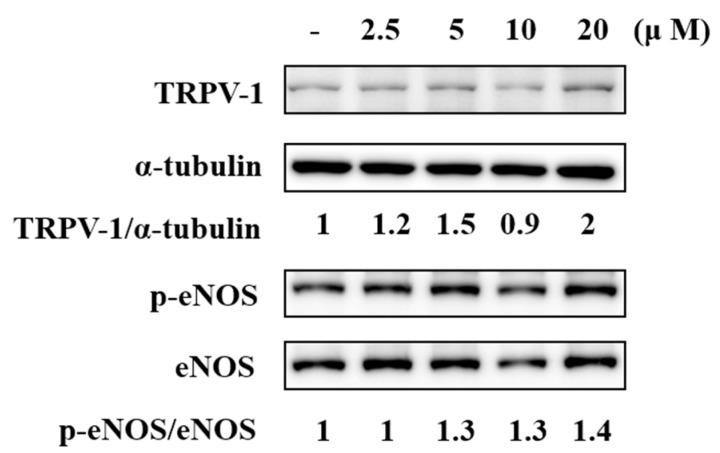
Effects of 10-fluoro-2-methoxyrutaecarpine (F-RUT) on transient receptor potential vanilloid-type 1 (TRPV-1) expression and endothelial nitric oxide synthase (eNOS) phosphorylation in human aortic endothelial cells (HAECs). The densitometric ratio is indicated.

TRPV1 is reportedly present in ECs of arteries. To validate the expression of TRPV1 in the endothelium, the TRPV1 protein of human aortic ECs (HAECs) was detected using immunoblotting. F-RUT treatment (20 μM) for 15 min increased TRPV1 protein amounts two-fold compared to the control group after normalization with α-tubulin levels (Figure 4). We further examined the effect of F-RUT on the phosphorylation of eNOS in HAECs because NO production is consequently regulated by the phosphorylation of eNOS. F-RUT treatment (20 μM) for 15 min significantly increased the phosphorylation of eNOS 1.4-fold compared to the control group after normalization with total eNOS (lower panel). F-RUT upregulated the expression of TRPV1 and activated eNOS phosphorylation in ECs.

### 2.6. F-RUT Ameliorates Inflammation in OVA/Alum-Challenged Mice

**Figure 5:**
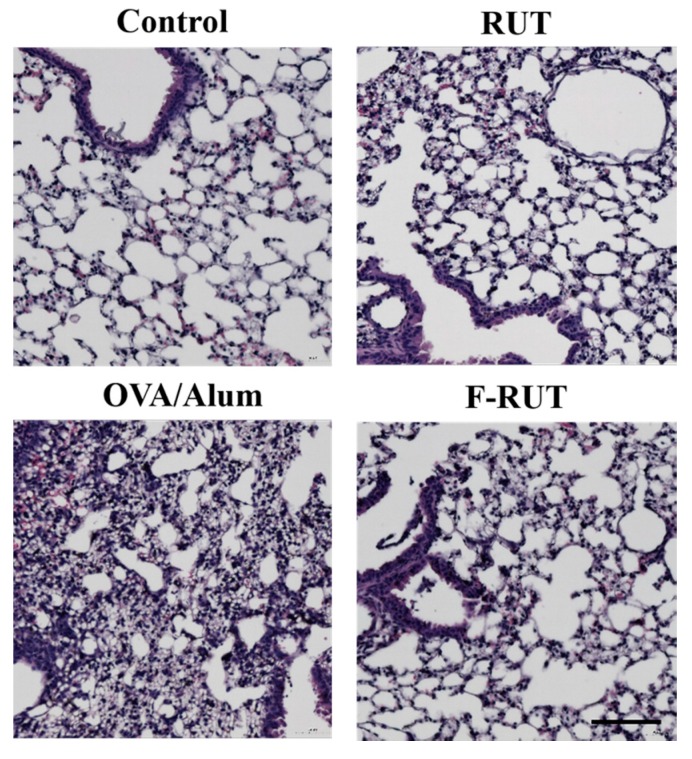
Effects of 10-fluoro-2-methoxyrutaecarpine (F-RUT) on ovalbumin (OVA)-challenged mice. Data are representative of three to five mice per group. Scale bar is 100 µm.

The anti-inflammatory effect of F-RUT was also examined in BALB/c mice. There was predominant inflammation in the lungs accomplished by increased infiltrating neutrophils after mice had been challenged with OVA/alum for 44 days. Alternate-day oral administration of RUT or F-RUT ameliorated the OVA/alum-induced lung inflammation and showed a similar pattern to the untreated control group (Figure 5).

## 3. Discussion

The anti-inflammatory activity of RUT was previously reported [7,29]. Vasodilator effects of RUT to induce CGRP synthesis and release were via activation of TRPV1. Therefore, its analogs were designed and synthesized for better vasodilator effects. Structural modifications of RUT were designed to enhance its biological activities. However, increased cytotoxicity hampers their application in vascular disorders [23,26,30]. F-RUT, a novel analog synthesized in this study with very low cytotoxicity showed anti-inflammatory activity and migration/invasion-suppressive activities that are beneficial in reducing side effects when used for pharmaceutics. eNOS and iNOS are isoforms with identical promoter elements that drive similar biological effects [31]. With respect to the diverse effects of F-RUT on eNOS and iNOS described in this study, they could have resulted from different signaling pathways in macrophages and ECs. F-RUT suppressed iNOS in macrophages, while it activated eNOS in ECs. The results bolster F-RUT, derived from RUT, having enhanced beneficial effects and reduced adverse effects.

OVA/alum-sensitized mice are a well-known animal model to induce lung inflammation [32,33,34]. In the airways, there are increased granulocytes, for example, neutrophils, and remodeling of the interstitium (capillary endothelium, alveolar epithelium, basement membrane, and perivascular tissue). In our experiment, F-RUT reduced infiltrating neutrophils and maintained the air sac structure in OVA/alum-challenged mice. These results might imply not just an anti-inflammatory effect but its benefit against damage due to remodeling between the epithelium and endothelium as well.

Hypertension activates pro-oxidant enzymes resulting in increased ROS formation, which is associated with Ang-II and mechanical forces, and damage to the vasculature [35]. Inflammation, migration, and fibrosis are important factors contributing to endothelial dysfunction and cardiovascular remodeling. Oxidative stress plays a physiological role in controlling endothelial function and also a pathophysiological role [36]. Many heart injuries result in fibrosis with deposition of excess collagens, or other matrix proteins, leading to the development of heart failure. Inflammation is the initial and primary trigger in cardiac stress and involves elevated levels of inflammatory cytokines and chemokines in tissues [37]. Fibrosis is characterized by the excess production of ECM produced by myofibroblasts. Studies showed that cardiac fibroblasts originate from the endothelial-to-mesenchymal transition (EndMT) of ECs [38], which is important in the formation of cardiac fibroblasts. The EndMT is regulated by signaling pathways mediated by inflammation-associated cytokines [39]. Direct contact with the bloodstream makes the endothelium a promising target for drug treatment. Ischemia/reperfusion injury leading to cardiac fibrosis is mainly mediated by collagen deposition by myofibroblasts. Snail induction is involved in fibrosis when undergoing the EndMT. Snail inhibitors remarkably suppressed collagen deposition and cardiac fibrosis in mice [40]. F-RUT treatment of A2780 cells produced reduced Snail protein levels (Supplemental Figure S8), which suggests that inhibition of the EndMT by F-RUT could be a new strategy for combating vascular diseases.

A previous study illustrated that inflammation and myofibroblast formation contribute to the development of pulmonary fibrosis [41]. Inflammatory cytokines induce the transformation of ECs to myofibroblasts through the EMT, and then produce excess ECM causing fibrosis. F-RUT, a derivative of RUT, possesses low cytotoxicity but retains its activities against inflammation and migration/invasion. Treatment with F-RUT enhanced TRPV1 and activated eNOS activity. According to the results, F-RUT might have potential applications in improving cardiac, vasodilation, and lung functions.

## 4. Materials and Methods

### 4.1. Chemicals and General Methods

All chemicals were purchased from Acros Organics (Geel, Belgium), Sigma-Aldrich (St. Louis, MO, USA), TCI America (Portland, OR, USA), and Showa Chemical Industry (Tokyo, Japan) without further purification. All reactions were processed in oven-dried glassware under an Ar or N_2_ atmosphere for anhydrous conditions. Analytical thin-layer chromatography (TLC) was performed on 0.2 mm-thick glass plate-mounted silica gel 60F254 (Merck KGaA, Darmstadt, Germany). Flash column chromatography was performed using Silicycle silica gel 60. Synthesized compounds were identified using Fourier-transformation infrared (FT-IR) spectroscopy (Bio-Rad Laboratories, Hercules, CA, USA) and ^1^H nuclear magnetic resonance (NMR; Bruker Avance 500 MHz, Billerica, MA, USA).

### 4.2. Synthesis of 10-Fluoro-2-methoxyrutaecarpine (F_MO_-RUT)

2-Amino-4,5-dimethoxybenzoic acid (**6**) (0.24 g, 1.5 mmol) was dissolved in toluene (5 mL) that cooled to 0 °C. Thionyl chloride (0.87 mL, 7.4 mmol) was added in a drop-wise manner to the ice-cold solution. The reaction mixture was heated to 70–80 °C and stirred for 1 h. The solution was heated to reflux after 10 min then cooled to 23 °C and concentrated under reduced pressure. The resulting residue was redissolved in toluene (5 mL), and the compound 2,3-piperidinedione-3-(4-fluorophenyl) hydrazone (**5**) (0.1 g, 0.5 mmol) was added. The reaction mixture was heated to reflux and stirred overnight. The solution was concentrated on a rotary evaporator, 10% sodium carbonate aqueous was added (200 mL), and the reaction was extracted by dichloromethane (3 × 200 mL). The organic layer was dried over anhydrous MgSO_4_, the solids were filtered through a fritted Büchner funnel, and the solution was concentrated under reduced pressure. The residue was purified by column chromatography (elution with EA:hexane = 1:2), affording F_MO_-RUT as a solid.

### 4.3. Cell Culture

The RAW264.7 macrophage cell line and A2780 ovarian carcinoma cells were grown in Dulbecco’s modified Eagle medium (DMEM) containing 10% fetal bovine serum (FBS), 100 U/mL penicillin, 100 μg/mL streptomycin, 1 mM sodium pyruvate, 4.5 g/L glucose, 4 mM l-glutamine, and 1.5 g/L sodium bicarbonate at 37 °C in a humidified atmosphere with 5% CO_2_. Primary human aortic ECs (HAECs) were grown in a MesoEndo Endothelial Cell Growth Medium Kit (Cell Applications, San Diego, CA, USA) supplemented with 10% FBS at 37 °C in a humidified atmosphere with 5% CO_2_.

### 4.4. Nitrite Assay

NO production was evaluated by measuring the nitrite concentration in supernatants of cultured RAW264.7 macrophages. Cells were first seeded at a density of 2 × 10^5^ cells/mL in 24-well plates for 24 h, followed by co-treatment with different concentrations of F-RUT with lipopolysaccharide (LPS) (40 ng/mL) for another 24 h. The amount of nitrite in cell culture supernatants was detected using the Griess reagent (1% sulfanilamide in 5% phosphoric acid and 0.1% naphthylethylenediamine dihydrochloride in water). Data are reported as the mean ± standard error of the mean (SEM) values of three independent determinations [42].

### 4.5. TNF-α Assay

Soluble cytokines were tested in supernatants of cultured RAW264.7 macrophages by an enzyme-linked immunosorbent assay (ELISA). Cells were plated at a density of 10^4^ cells/mL in 96-well plates for 12 h, followed by treatment with different concentrations of F-RUT for 1 h and then treatment with LPS (40 ng/mL) for 24 h. TNF-α in cell supernatants was detected using a mouse TNF-α Quantikine kit (R&D Systems, Minneapolis, MN, USA) according to the manufacturer’s instructions. The absorbance was read at 450 nm with an ELISA plate reader. Data were reported as the mean ± SEM values of three independent determinations.

### 4.6. Cell Viability Assay

An MTT assay to test cell viability was performed as described previously, based on the conversion of the yellow tetrazolium salt to the purple formazan product [43]. Cells (10^4^ cells/well) were grown in a 96-well plate supplemented with standard culture medium. Cells were treated with RUT and F-RUT (0–20 μM) for 24 h. An MTT stock solution (5 mg of MTT/mL of phosphate-buffered saline; PBS) was added to the growing cultures for 2 h. The absorbance was measured with a spectrophotometer at 560 nm. DMSO alone was measured as a reading control. Data were reported as the mean ± SEM values of five independent determinations.

### 4.7. Western Blot Analysis

Protein samples were separated and resolved by sodium dodecylsulfate polyacrylamide gel electrophoresis (SDS-PAGE) and electrotransferred onto a polyvinylidene difluoride (PVDF) membrane. The membrane was incubated with a primary antibody at 4 °C overnight, and then incubated with a horseradish peroxidase (HRP)-conjugated secondary immunoglobulin G (IgG) antibody; immunoreactive bands were visualized with PerkinElmer enhanced chemiluminescent reagents [44].

### 4.8. Transient Transfection and Luciferase Assay

RAW264.7 macrophages were seeded in a 96-well plate with DMEM. Then, cells were transfected with the pGL4.32 [luc2P/NF-κB-RE/Hygro] (Promega, Madison, WI, USA) plasmid reporter gene using TurboFect Transfection Reagent (Fermentas, Glen Burnie, MD, USA). At 24 h after transfection, cells were treated with LPS (40 ng/mL) and F-RUT for 24 h in serum-free medium. Then the luciferase activity was detected by the luminescence measured in a luminescence microplate reader (Thermo Varioskan Flash, Waltham, MA, USA) using a ONE-Glo luciferase assay kit (Promega). Luciferase activities were normalized to protein concentrations.

### 4.9. Wound-Healing Migration Assay

A2780 cells were cultured at a density of 2 × 10^5^ cells/well in 6-well plates and incubated at 37 °C for 24 h. A centerline in the cells was scratched using a 200-μL pipette tip and washed with PBS. Then, new complete medium was added and treated with or without 1 and 2.5 μM of F-RUT for 24 h. At the endpoint of incubation, cells were examined and photographed with an optical microscope. The distance between the edges of the scratched area was measured and calculated to estimate the migratory ability of cells [45].

### 4.10. In Vitro Invasion Assays

A2780 cell invasion was evaluated using 24-well transwell inserts (8-μm-pore filters, Merck Millipore) individually coated with Matrigel (BD Biosciences, Bedford, MA, USA). A2780 cells (2 × 10^4^ cells in each well) were cultured for 24 h with serum-free minimum essential medium (MEM) and then treated with F-RUT (1.25 or 2.5 μM) for another 24 h in the upper chamber of the transwell. Medium containing 10% FBS was placed in the lower chamber. At the end of incubation, non-migrated cells were removed using a cotton swab; cells that had penetrated to the opposite surface of the filter were fixed with 4% formaldehyde and stained with 2% crystal violet. Stained cells were counted and photographed under a phase-contract optical microscope at 200× magnification. Three independent experiments were performed as described elsewhere [45].

### 4.11. Animal Experiment

BALB/c mice (six weeks of age) were obtained from the Animal Center of the College of Medicine, National Taiwan University (Taipei, Taiwan), and sensitized with an intraperitoneal injection of 20 μg of ovalbumin (OVA) emulsified in 2 mg of aluminum hydroxide in a total volume of 200 μL phosphate-buffered saline (PBS) on day 0, and boosted with 50 μg of OVA emulsified in 4 mg of aluminum hydroxide on days 14 and 28. RUT or F-RUT was given by oral administration on days 30, 32, 34, 36, and 38. For post-challenge, all mice were treated intranasally with OVA (100 μg in a total volume of 40 μL PBS) on days 40, 41, 42, and 43. At 24 h after the last OVA challenge, mice were sacrificed, and their organs were collected. All experimental procedures were reviewed and approved by the Institutional Animal Care and Use Committee or Panel. Lung tissues were fixed in 4% paraformaldehyde (sc-281692; Santa Biotechnology) and embedded in paraffin. Tissue sections were made at a 5-μm thickness, and stained with hematoxylin and eosin (H&E) solution for examination of inflammation [34].

## Supplementary Materials

Supplementary materials are available online.
