# Supplementary material for: Synthetic Fluororutaecarpine Inhibits Inflammatory Stimuli and Activates Endothelial Transient Receptor Potential Vanilloid-Type 1
Source: Molecules. 2017 Apr 19;22(4):656. doi: 10.3390/molecules22040656 (PMC6153741; doi:10.3390/molecules22040656)
Supplement: Supplementary file 1 [file molecules-22-00656-s001.pdf]

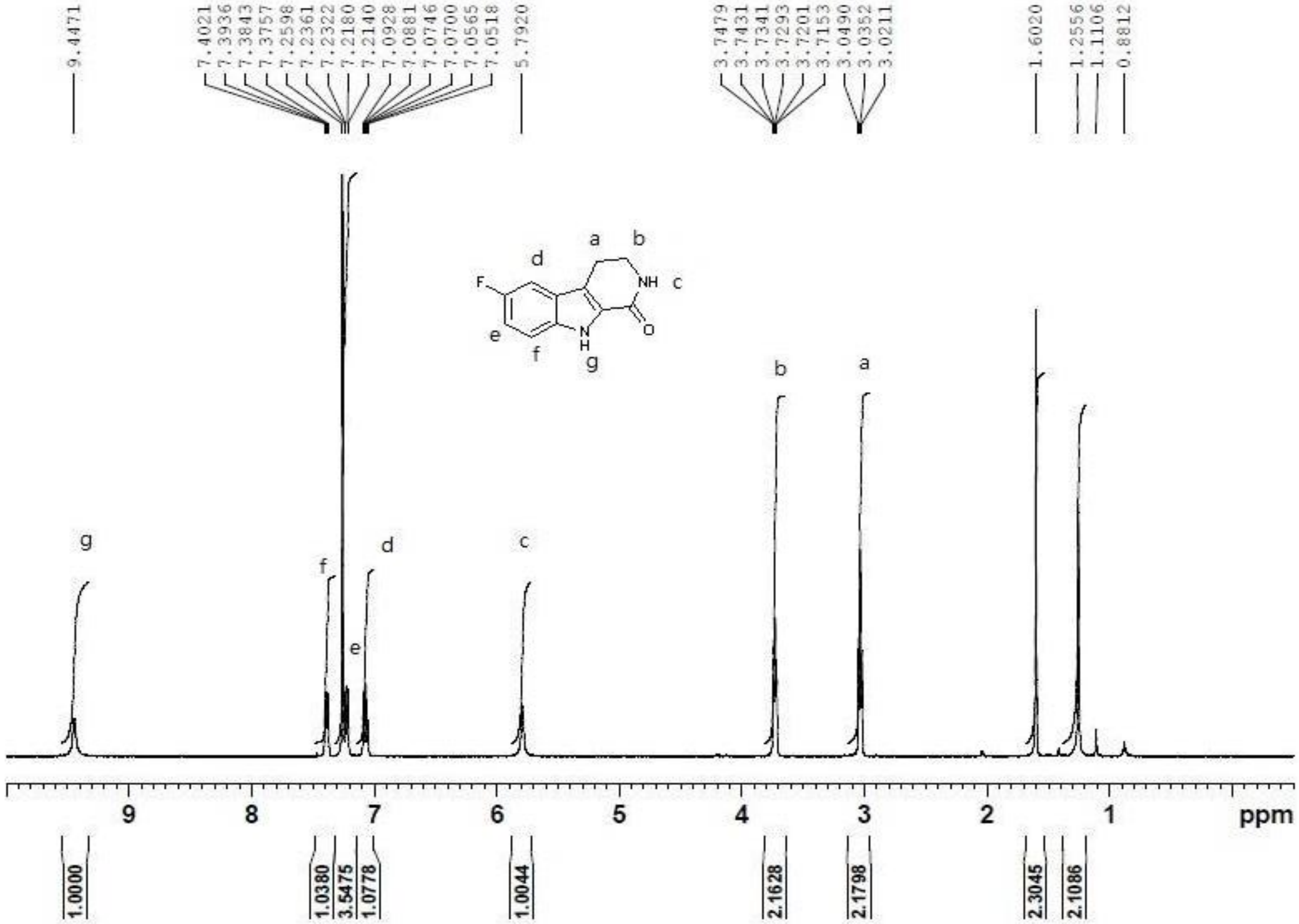

Fig. S1. ^{1}H NMR spectrum of compound 5

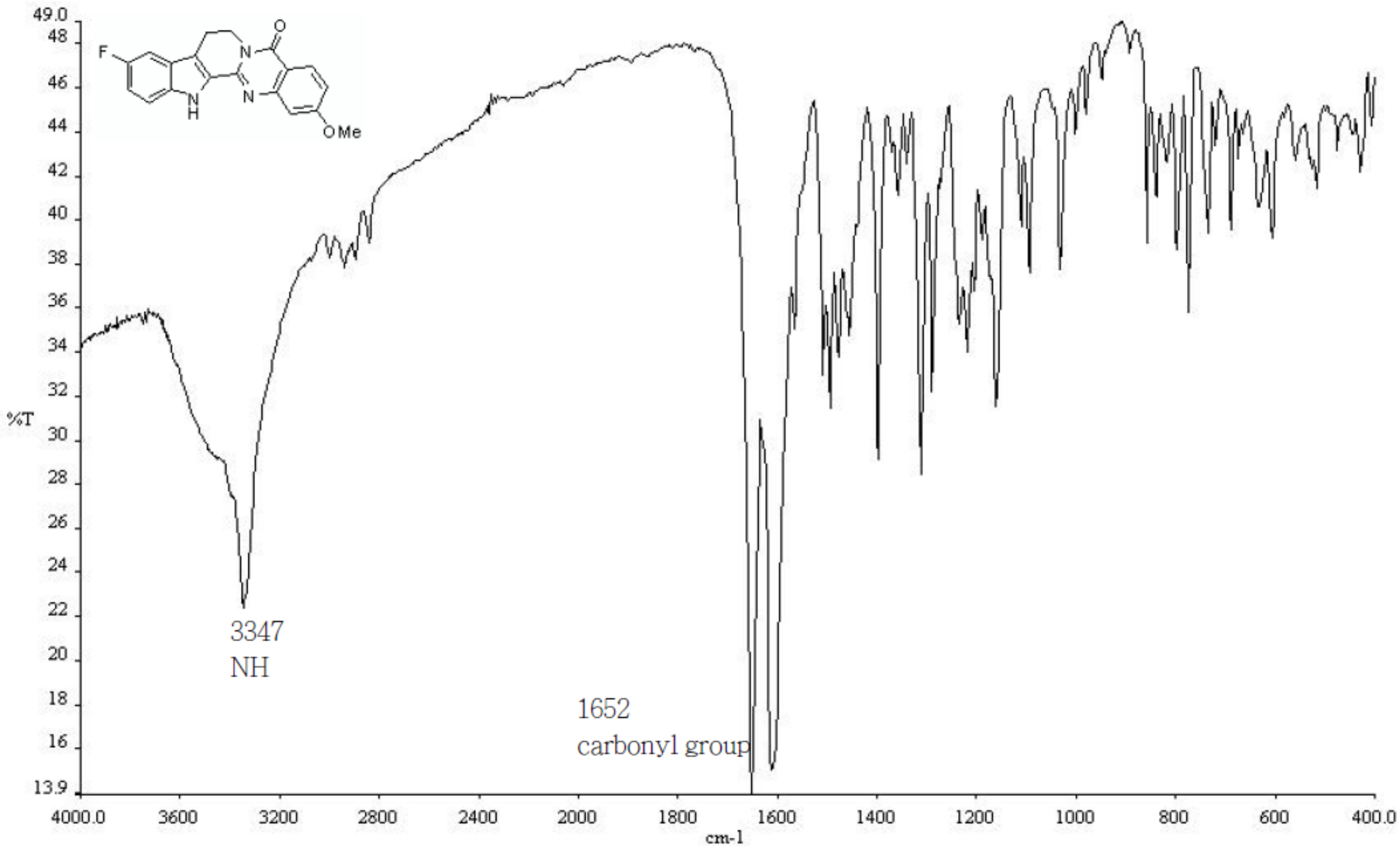

Fig. S2. FT-IR (KBr, cm-1) spectrum of F_{mo}-RUT

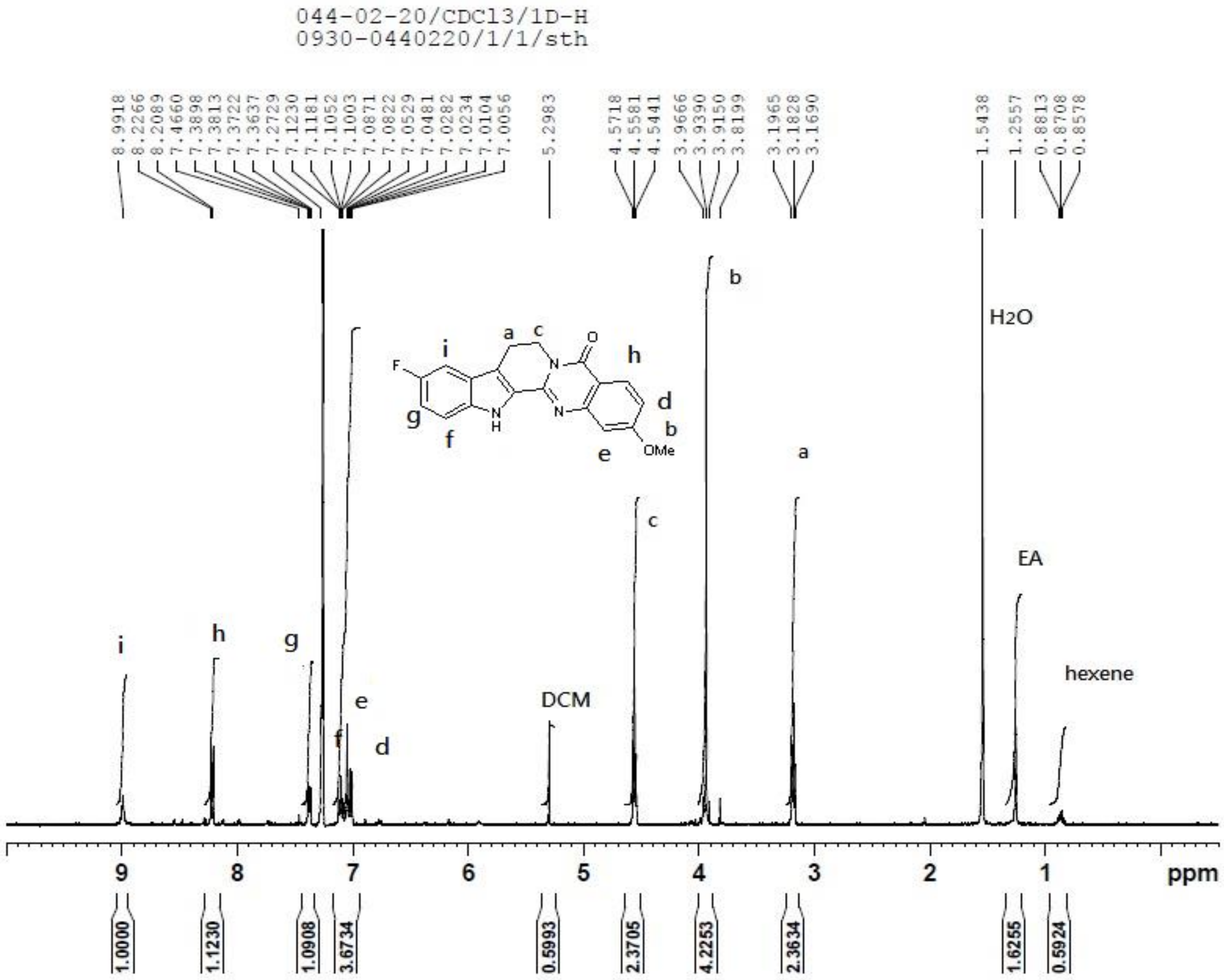

Fig. S3. ^{1}H NMR spectrum of F_{mo}-RUT

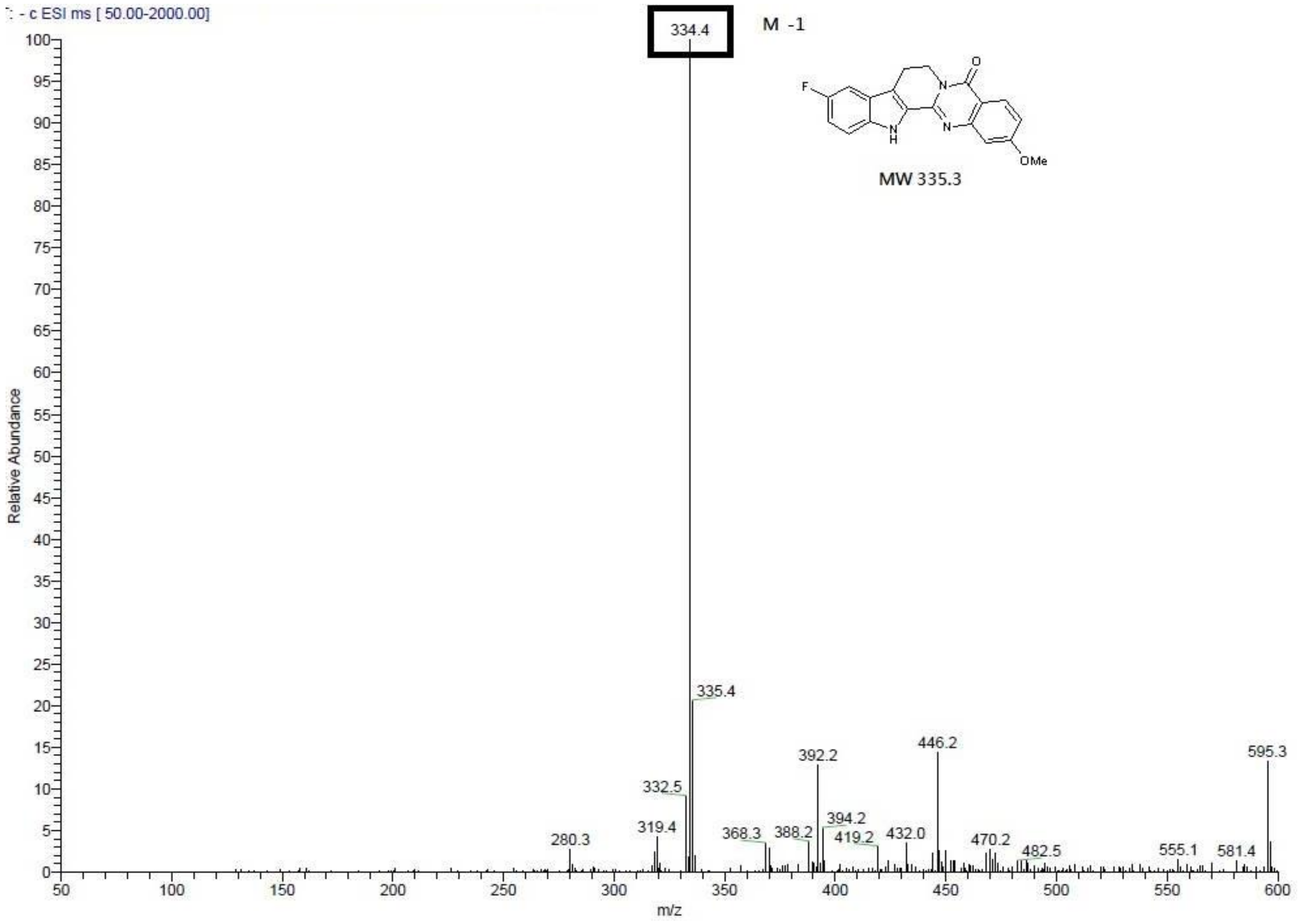

Fig. S4. MS-EI (m/z) spectrum of F_{mo}-RUT

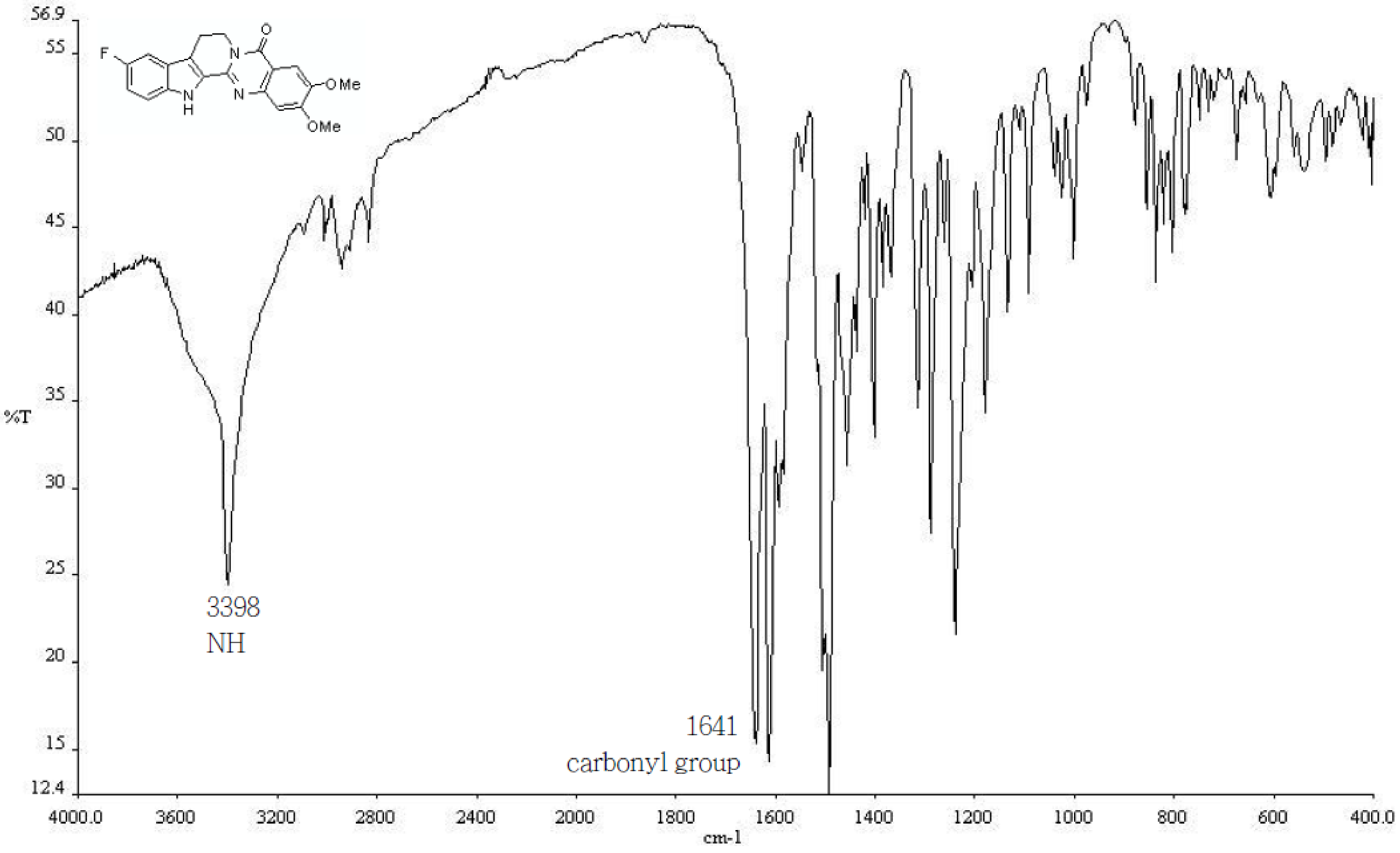

Fig. S5. FT-IR (KBr, cm-1) spectrum of F_{2mo}-RUT

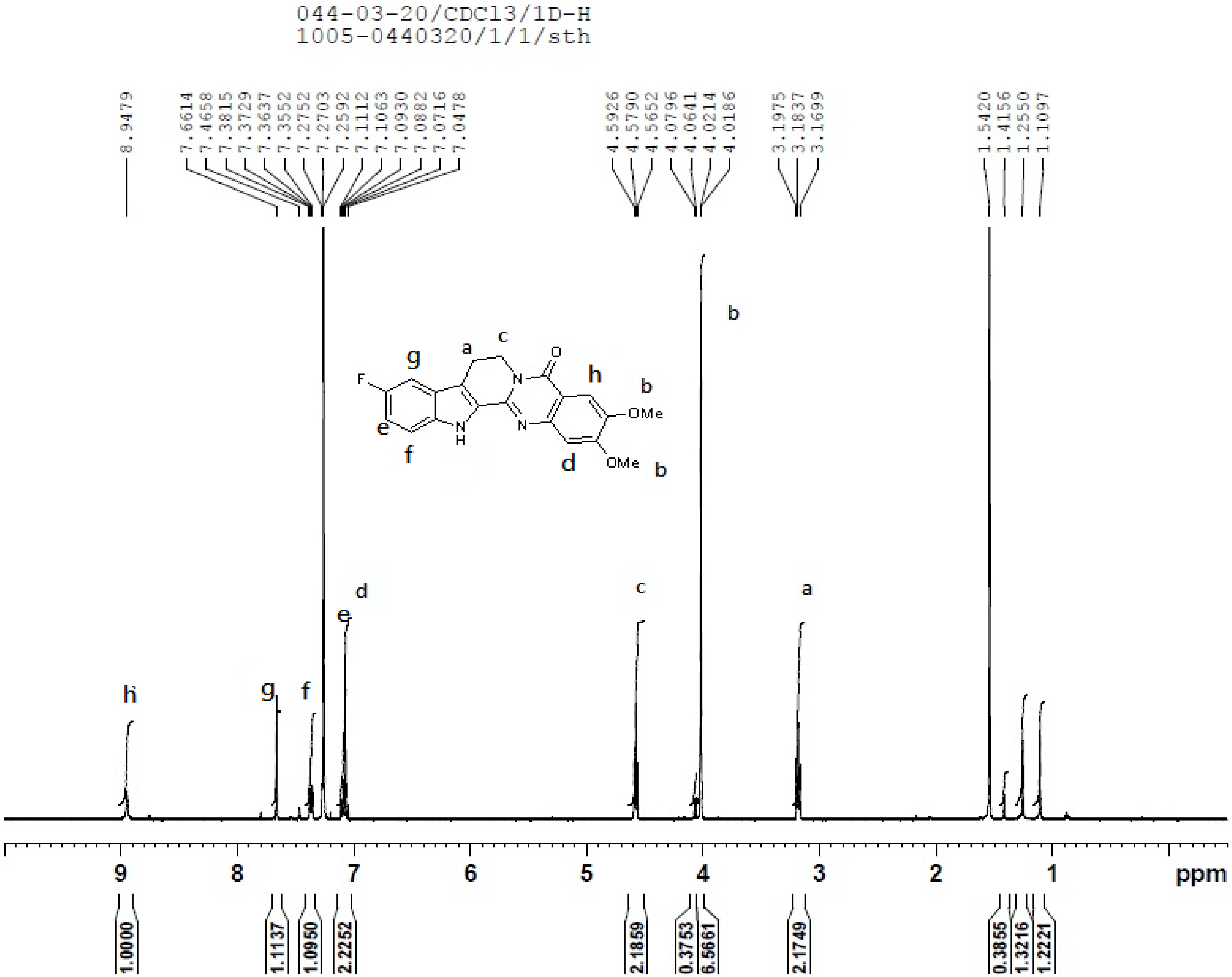

Fig. S6. ^{1}**H NMR spectrum of** F_{2mo}**-RUT**

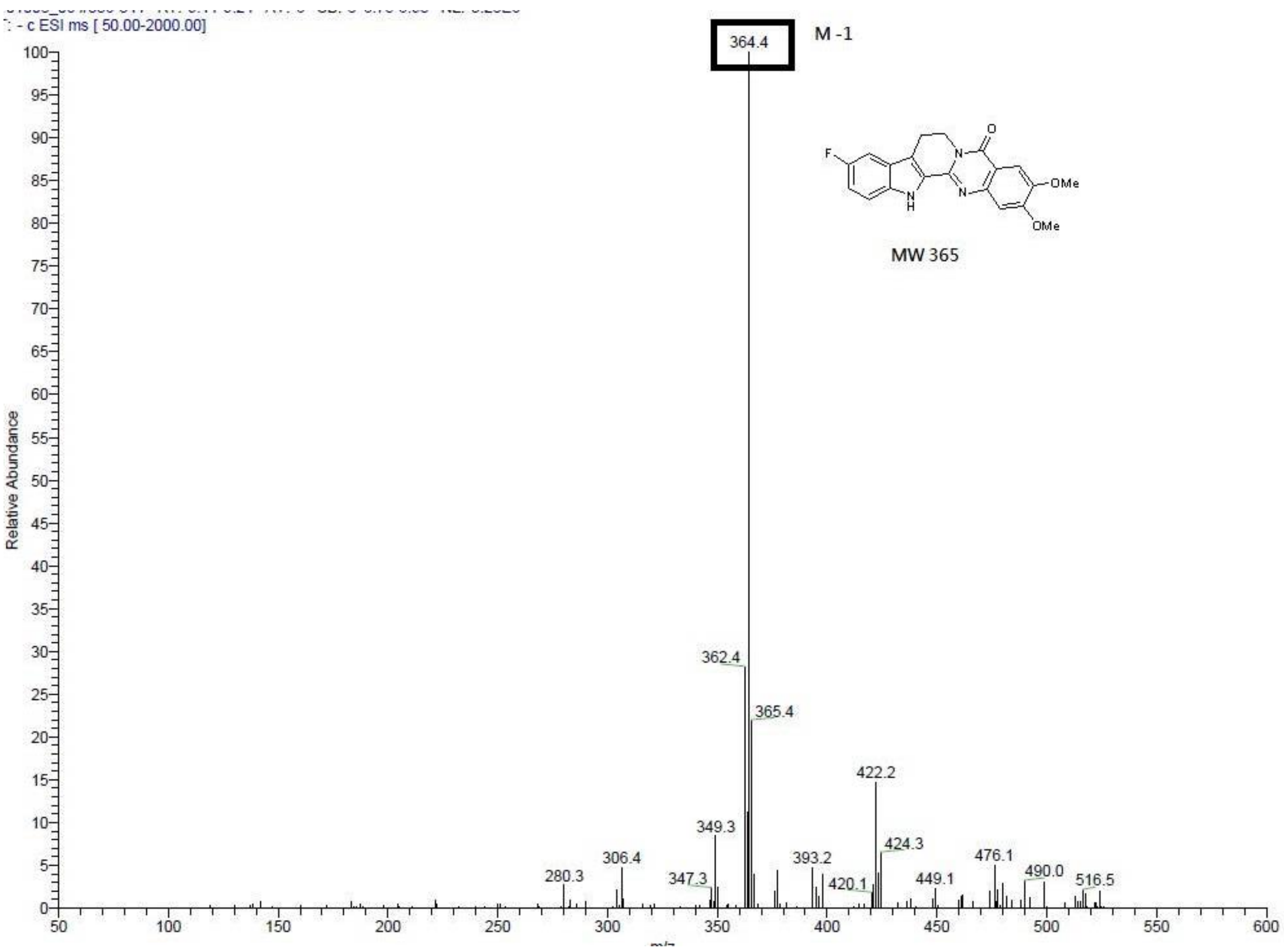

Fig. S7. MS-EI (m/z) spectrum of F_{2mo}-RUT

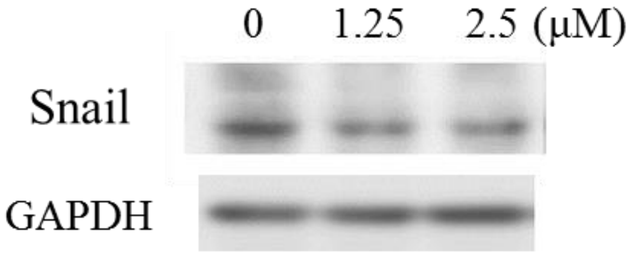

Fig. S8. Snail expression of F-RUT (0-2.5 μM)-treated A2780 cells
